# Active Component of Danshen (*Salvia miltiorrhiza* Bunge), Tanshinone I, Attenuates Lung Tumorigenesis via Inhibitions of VEGF, Cyclin A, and Cyclin B Expressions

**DOI:** 10.1155/2013/319247

**Published:** 2013-04-09

**Authors:** Yu-Tang Tung, Hsiao-Ling Chen, Cheng-Yu Lee, Yu-Ching Chou, Po-Ying Lee, Hsin-Chung Tsai, Yi-Ling Lin, Chuan-Mu Chen

**Affiliations:** ^1^Department of Life Sciences, Agricultural Biotechnology Center, National Chung Hsing University, Taichung 402, Taiwan; ^2^Department of Bioresources and Molecular Biotechnology, Da-Yeh University, Changhwa 515, Taiwan; ^3^Department of Plant Industry, National Pingtung University of Science and Technology, Pingtung 912, Taiwan; ^4^Department of Surgery, National Taiwan University Hospital Yun-Lin Branch, Yun-Lin 640, Taiwan; ^5^Taichung Hospital, Department of Health, Taichung 403, Taiwan; ^6^Department of Surgery, Taichung Veterans General Hospital, Taichung 407, Taiwan

## Abstract

Tanshinone I (T1) and tanshinone II (T2) are the major diterpenes isolated from Danshen (*Salvia miltiorrhiza* Bunge). Three human lung adenocarcinoma cell lines, A549, CL1-0, and CL1-5, were treated with T1 and T2 for the *in vitro* antitumor test. Results showed that T1 was more effective than T2 in inhibiting the growth of lung cancer cells via suppressing the expression of VEGF, Cyclin A, and Cyclin B proteins in a dose-dependent manner. Moreover, a transgenic mice model of the human vascular endothelial growth factor-A_165_ (*hVEGF*-*A*_165_) gene-induced pulmonary tumor was further treated with T1 for the *in vivo* lung cancer therapy test. T1 significantly attenuated hVEGF-A_165_ overexpression to normal levels of the transgenic mice (Tg) that were pretreated with human monocytic leukemia THP-1 cell-derived conditioned medium (CM). It also suppressed the formation of lung adenocarcinoma tumors (16.7%) compared with two placebo groups (50% for Tg/Placebo and 83.3% for Tg/CM/Placebo; *P* < 0.01). This antitumor effect is likely to slow the progression of cells through the S and G2/M phases of the cell cycle. Blocking of the tumor-activated cell cycle pathway may be a critical mechanism for the observed antitumorigenic effects of T1 treatment on vasculogenesis and angiogenesis.

## 1. Introduction

According to statistics supplied by the Taiwanese Department of Health, pulmonary cancer is the most common invasive malignancy and the leading cause of cancer deaths in Taiwan [1]. Pulmonary cancer, according to the biocharacteristics and the clinical manifestation, can be divided into two gross types including small cell lung cancer and nonsmall cell lung cancer. According to the statistics of epidemiology in Taiwanese district, among patients with lung cancers, the ratio of patients of small cell lung cancer is only 12%–15% and the ratio of patients of nonsmall cell lung cancer is about 85%–88%. The nonsmall cell lung cancer primarily includes pulmonary squamous cell carcinoma, pulmonary large cell lung carcinoma, and pulmonary adenocarcinoma [2]. Pulmonary adenocarcinoma induces a tumor resulted from cells classified as secretory cells including Clara cell, type II alveolar cells, and mucin producing cells. Pulmonary adenocarcinoma commonly occurs in the peripheral of lung (about 2/3) and the other 1/3 of it starts proliferation from the center of lung [3]. Upon forming a tumor by pulmonary adenocarcinoma in a patient, it will cause distal metastasis to other organs including brain, kidney, liver, and bone in 80% of patients. Therefore, many researchers have searched for more effective treatments to cure pulmonary adenocarcinoma [4].

In the early stages of cancer, the out-of-control proliferation of cancer cells leads to a deficiency of both nutrients and oxygen that causes a large degree of cell death. Therefore, an inflammation response occurs, and hypoxia-inducible factor-1*α* (HIF-1*α*) is activated. The HIF-1*α* activation induces secretion of a large quantity of vascular endothelial growth factor-A_165_ (VEGF-A_165_). The secreted VEGF-A_165_ binds to VEGFR2 receptor and activates a downstream signal that induces vasculogenesis [5, 6]. When cancer cells secrete a large amount of VEGF-A_165_, vasculogenesis is induced so as to provide sufficient nutrition and oxygen to the tumor, thus increasing tumor growth rate. It is well known that the expression level of VEGF-A_165_ is positively related to the growth and spread of cancer cells [7]. Therefore, the development of medicines that target VEGF-A_165_ is a popular topic of study.

Traditional medicine usually contains various bioactive phytochemicals with the chemoprevention and therapy of cancer [8]. Danshen (*Salvia miltiorrhiza* Bunge) is a traditional medicine that has been used in China for over a thousand years to treat various diseases, including coronary artery disease, cerebrovascular disease, heart disease, hepatitis, and cancer [9–11]. Over 40 tanshinone compounds have been isolated and identified from Danshen. Of these, tanshinone I (T1), tanshinone II (T2), and cryptotanshinone (CTS) are the three major diterpene compounds [11, 12], which possess anticancer properties [8]. Furthermore, T1 reduced the growth of leukemia [13–16], lung cancer [17], and breast cancer [18] in *in vitro* cell cultures via induction of apoptosis.

In this study, an expression vector carrying a mouse *Clara cell secretory protein (mCCSP)* gene promoter and the human *vegf*-*A*_165_ gene cDNA sequence was constructed. Then, the expression vector was introduced into the embryos of FVB mice by a microinjection process and the embryos were transplanted into the fallopian tubes of female mice. The embryos are allowed to develop into the newborn mice and the hVEGF-A_165_ is capable of expressing in the lung bronchus epidermal cells of the transgenic mice. The transgenic mouse carrying *h* *vegf*-*A*_165_ gene, which induces pulmonary tumor, was used as an *in vivo* lung cancer therapy model.

T1 had antitumorigenic and antimetastatic effects in CL1-5-bearing SCID mice when coinjected with condition medium (CM) which is a serum-free medium with 24 h incubation with phorbol myristate acetate-treated human monocytic leukemia THP-1 cells [17]. Previous studies showed that the CM contains proinflammatory cytokines such as TNF-*α*, IL-1, IL-8, and IL-12; anti-inflammatory cytokines such as TGF-*β*, IL-4, IL-10, and IL-13; and IL-6 with both proinflammatory and anti-inflammatory properties [19–23]. Previous reports have shown that the imbalance between proinflammatory and anti-inflammatory cytokines can influence neoplastic outcome [19]. Lee et al. [17] showed that the CM treatment alone would invoke inflammation and neovascularization and promote tumor growth due to the abundant proinflammatory cytokines. Thus, in this study a strain of human *h* *vegf*-*A*_165_-induced lung tumorigenic transgenic mice was used to research the regulatory mechanism of T1 coinjected with CM against pulmonary adenocarcinoma.

## 2. Methods

### 2.1. Chemicals

**Figure 1:**
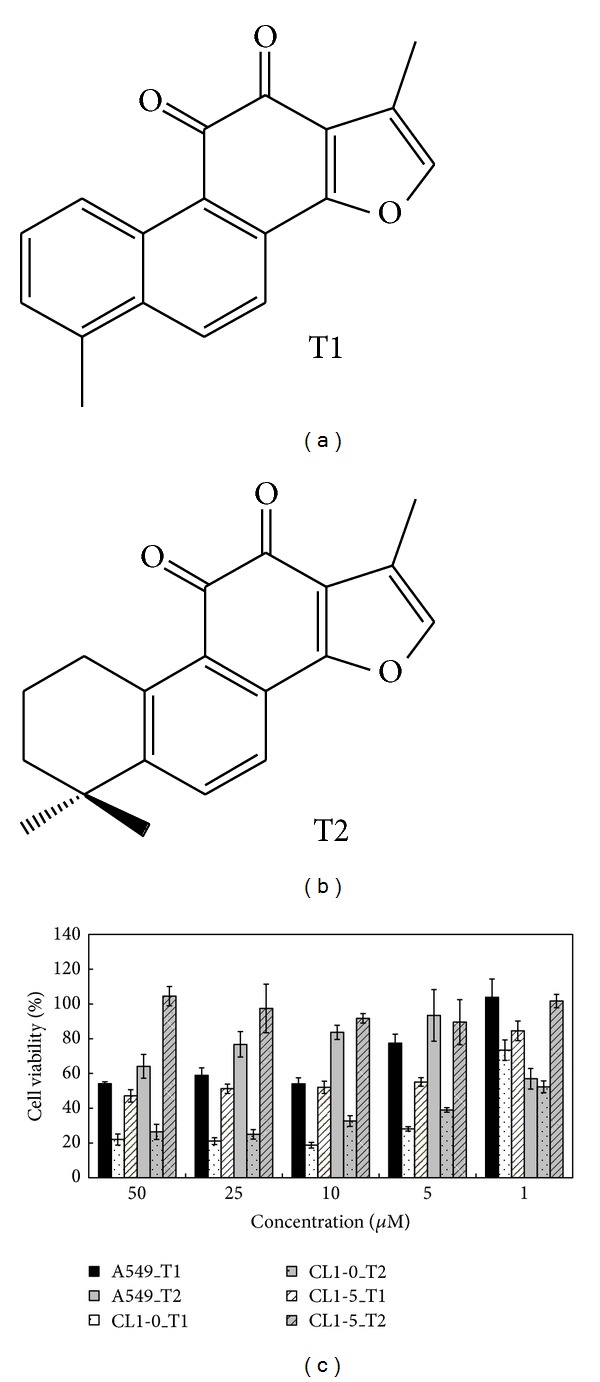
Active components of tanshinone I (T1) and tanshinone II (T2) extracted from a Chinese traditional medicine, Danshen (*Salvia miltiorrhiza *Bunge), and the cell viability of human lung cancer cell lines after T1 and T2 treatments. (a) Chemical structure of T1. (b) Chemical structure of T2. (c) Effects of T1 and T2 over a range of concentrations (1, 5, 10, 25, and 50 *μ*M) on the cell viability of A549, CL1-0, and CL1-5 cells after 24 h incubation.

Tanshinone I (1,6-dimethyl-phenanthro [1,2-*b*]furan-10,11-dione) (purity 99%) and tanshinone II (1,6,6-trimethyl-6,7,8,9-tetrahydrophenanthro [1,2-*b*]furan-10,11-dione) (purity 99%) were obtained from Formosa Kingstone Bioproducts International (Taipei, Taiwan) (Figure 1). Tanshinones were isolated from the root of *S. miltiorrhiza* Bunge.

### 2.2. Cell Line

The human monocytic leukemia cell line THP-1 (ATCC TIB 202; American Type Culture Collection) was grown in RPMI 1640 (Invitrogen Corp., Carlsbad, CA, USA) supplemented with 1.5 g/L Na_2_HCO_3_, 4.5 g/L glucose, and 10% fetal bovine serum (FBS). The cell line was incubated at 37°C in 5% CO_2_. Before the experiments, THP-1 cells were pretreated with 3.2 × 10^7^ mole/L phorbol myristate acetate (Sigma-Aldrich Corp., St. Louis, MO, USA) for 24 h. The preparation of conditioned medium (CM) derived from phorbol myristate acetate-pretreated THP-1 cells has been described previously [24]. Human lung adenocarcinoma cell lines, A549, CL1-0, and CL1-5 cells, were cultured in Dulbecco's modified Eagle's medium (DMEM) supplemented with 10% FBS. Cells were incubated at 37°C in 5% CO_2_.

### 2.3. Cell Viability on Tumor Cells by MTT Assay

To measure the cytotoxicity of tanshinone I and tanshinone II on cell proliferation, A549, CL1-0, and CL1-5 cells (2 × 10^5^ cells/well) were seeded into a 96-well plate in triplicate and preincubated for 3 h to allow cell adherence. First, 200 *μ*L of fresh medium containing various concentrations (1, 5, 10, 25, and 50 *μ*M) of tanshinone I and tanshinone II was added into the cultures and incubated at 37°C for 24, 48, and 72 h under humidified air containing 5% CO_2_. Following the removal of the medium from the wells, 100 *μ*L of tetrazolium salt solutions (1 mL MTT in 10 mL DMEM) was added. After 4 h of incubation at 37°C, the medium was removed and 100 *μ*L of DMSO was added to dissolve the formazan crystals. Absorbance was measured in an enzyme-linked immunosorbent assay (ELISA) reader at 570 nm. The cell viability ratio (%) was calculated from the following equation: % viability = (absorbance of test sample/absorbance of control) × 100.

### 2.4. Transgenic Mouse Production and Validation

The mccsp-hVEGF-A_165_-sv40 transgenic mice were generated by pronuclear microinjection. A 1975-bp transgene fragment, consisting of *mCCSP* promoter, *hVEGF*-*A*_165_ cDNA, and SV40 poly(A) signal sequence, was obtained from the plasmid with *Nru*I-*Sap*I double-digestion. The purified transgene was microinjected into the male pronuclei of fertilized eggs from superovulated female mice and transferred to recipient pseudopregnant females [22]. To detect the hVEGF-A_165_ transgene in transgenic mice with a homozygous (hVEGF-A_165_^+/+^) or heterozygous (hVEGF-A_165_^+/−^) genotype, the mice were rapidly screened for the foreign gene by PCR analysis [25]. The exogenic human VEGF-A_165_ protein expression levels in homozygous (hVEGF-A_165_^+/+^) or heterozygous (hVEGF-A_165_^+/−^) transgenic mice were also detected by western blot [25]. In this study, we used the homozygous (hVEGF-A_165_^+/+^) genotype to evaluate the anticancer effects of T1.

### 2.5. Animals

The transgenic mice and wild-type FVB strain mice were sustained on a standard laboratory diet and distilled water *ad libitum* and kept on a 12-hour light/dark cycle at 22 ± 2°C. This study was conducted according to institutional guidelines and approved by the Institutional Animal Care and Utilization Committee of National Chung Hsing University, Taiwan (IACUC No. 96-83). For the examination of both *VEGF* expression and pulmonary function, the transgenic mice with the homozygous genotype (hVEGF-A_165_^+/+^) were normally distributed to three groups (*n* = 6) and treated as follows: (1) PBS alone (Tg/Placebo group), (2) CM alone (Tg/CM/Placebo group), and (3) T1 (1 mg/kg body weight) suspended in CM (Tg/CM/T1 group). Three groups were injected intraperitoneally (*i.p.*) three times a week. In addition, wild-type FVB strain mice (*n* = 6) were used as a normal control group. Experimental mice were sacrificed at the age of 10 months old after stimulation by CM during months 2–4 and 6–10 and then receiving T1 or placebo administration at months 6–10. Pulmonary tissues were collected for pathological histology, immunohistochemical (IHC) staining [26, 27], and RNA [28] and protein extraction.

### 2.6. Measurement of Airway Hyperresponsiveness (AHR)

To evaluate AHR in the Tg/Placebo, Tg/CM/Placebo, and Tg/CM/T1 groups, bronchial provocation tests were performed using methacholine. First, the basal pulmonary function was measured; then, saline and methacholine (12.5 mg/mL) were converted to aerosols using a nebulizer; finally, the mice inhaled the aerosolized compounds five times through a Rosenthal-French dosimeter. Three minutes later, a pulmonary function test was performed. The pulmonary function at each step was measured 30 times with a portable microspirometer, and the enhanced pause (penh) values were selected as the pulmonary function value [25].

### 2.7. Pathological Histology

Lung tissue was fixed in 10% buffered formaldehyde (pH 7.0), embedded with paraffin, sectioned into 3 *μ*m sections, and examined using hematoxylin and eosin (H&E) staining as described previously [29, 30].

### 2.8. Immunohistochemistry (IHC) Staining

Formaldehyde-fixed and paraffin-embedded sections were cut to a thickness of 5 *μ*m, deparaffinized and rehydrated in a gradient of alcoholic solutions, and then treated with boiling water for 15 minutes. The sections were incubated in 3% hydrogen peroxide for 10 min to block endogenous peroxidase activity and then incubated overnight at 4°C with primary rabbit monoclonal antibody against hVEGF-A using a 1 : 40 working dilution. For antigen retrieval, the sections were immunostained with the VECTASTAIN ABC kit (Universal, Vector Lab., Burlingame, CA, USA) in accordance with the manufacturer's specifications. Diaminobenzidine (DAB) was used for stain development, and the sections were counterstained with hematoxylin [31]. The negative control consisted of substituting normal serum for primary antibody.

### 2.9. Real-Time RT-PCR

Total RNA was extracted from lung tissue using Trizol reagent (Invitrogen, Carlsbad, CA, USA), as specified by the manufacturer. The total RNA (2 *μ*g) was then resuspended in 9 *μ*L of diethylpyrocarbonate-(DEPC-) treated water, and the first strand of cDNA was synthesized with random primers using ImProm-II reverse transcriptase (Promega, USA) in a total volume of 20 *μ*L. The reaction was carried out at 42°C for 1 h. For further PCR amplification, an aliquot (1 : 10) of the RT product was adjusted to contain 0.1 *μ*g of each primer, and additional buffer was added to a total volume of 20 *μ*L. RT-PCR was performed in a Thermal Cycler 2720. Real-time RT-PCR was performed using SYBR Green in a Rotor-Gene 6000 [26]. To evaluate gene expression, real-time RT-PCR was performed on 6 genes (*nrp-1*, *kdr*, *mmp2*, *egfr*, *erk2,* and *survivin*) using cDNA from pulmonary tissue. The cDNA of **β*-actin *was used as an internal control.

### 2.10. Western Blotting

CL1-0 cells were seeded on a 10 cm dish for 2 h; afterward, the cells were pretreated with T1 (1, 5, 10, and 25 *μ*M) for 24 h. The expressions of VEGF, Cyclin A, and Cyclin B proteins were measured by western blotting. Pulmonary tissues were homogenized in 500 *μ*L of RIPA buffer (5 mM Tris-HCl pH 7.4, 0.15 M NaCl, 1% NP40, 0.25% sodium deoxycholate, 5 mM EDTA, and 1 mM ethylene glycol-bis (2-aminoethyl-ether)-N, N, N, N-tetraacetic acid). The homogenates were centrifuged at 12,000 g for 30 minutes at 4°C. Protein (40 *μ*g) was then separated by SDS-PAGE in 10% polyacrylamide and electrotransferred to polyvinylidene difluoride membranes. The membranes were incubated in blocking solution (5% BSA) at room temperature for 2 h. The membranes were then incubated with primary antibody (VEGF-A, ERK2, Cyclin A, Cyclin B, and GAPDH) overnight at 4°C. After washing, the membranes were incubated with a goat anti-rabbit IgG peroxidase-conjugated secondary antibody directed against the primary antibody. The membranes were developed using an enhanced chemiluminescence western blot detection system as described previously [32].

### 2.11. Statistical Analysis

Experimental values are expressed as the mean ± standard error (SE). All data were analyzed using *t*-tests. Statistical significances are presented as *P* < 0.05 (*) or *P* < 0.01 (**).

## 3. Results

### 3.1. Effects of T1 and T2 on Cancer Cell Viability

A549, CL1-0, and CL1-5 cells, which are human lung adenocarcinoma cell lines, were treated with the major active components isolated from Danshen, tanshinone I (T1; Figure 1(a)) and tanshinone II (T2; Figure 1(b)) at concentrations of 1, 5, 10, 25, and 50 *μ*M for 24, 48, and 72 h. This treatment decreased the cell viability in a concentration-dependent manner when compared with controls (Figure 1(c)). In MTT assay, T1 was more effective than T2, and the IC_50_ values of T1 were 8.9, 1.8, and 2.0 *μ*M for A549, CL1-0, and CL1-5 cells at 24 h, respectively. Of these cancer cells, T1 was able to successfully inhibit the growth of CL1-0 cells.

### 3.2. T1 Suppressed VEGF, Cyclin A, and Cyclin B Protein Expressions in CL1-0 Lung Cancer Cells

**Figure 2:**
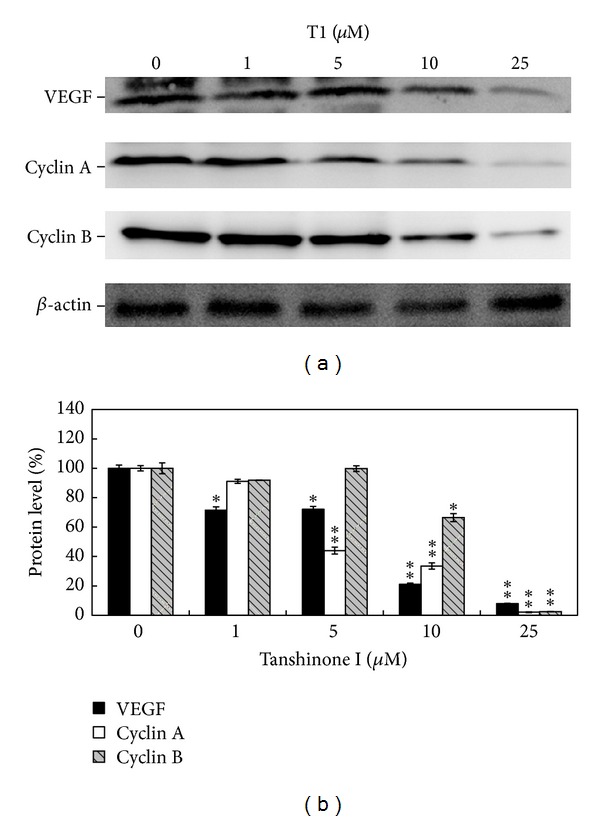
Expressions of VEGF, Cyclin A, and Cyclin B protein in CL1-0 cells treated with T1 after 24 h incubation. (a) Western blot analysis of VEGF, Cyclin A, and Cyclin B protein expressions in CL1-0 cells after being treated with different concentrations of T1 (0, 1, 5, 10, and 25 *μ*M) for 24 h. (b) The quantification data of protein expression levels under different concentrations of T1 treatment.

As shown in Figure 2(a), T1 inhibited the expressions of VEGF, Cyclin A, and Cyclin B protein in a dose-dependent manner. Approximately 77%, 60%, and 25% reductions of VEGF, Cyclin A, and Cyclin B protein were observed at 10 *μ*M, as determined with densitometry analysis (Figure 2(b)). At concentrations of up to 10 *μ*M, T1 could almost completely inhibit the expression of Cyclin A, and Cyclin B proteins in CL1-0 cells. Thus, T1 warrants further development as a cancer-prevention agent.

### 3.3. Effect of T1 on AHR Parameters of Pulmonary Function

**Figure 3:**
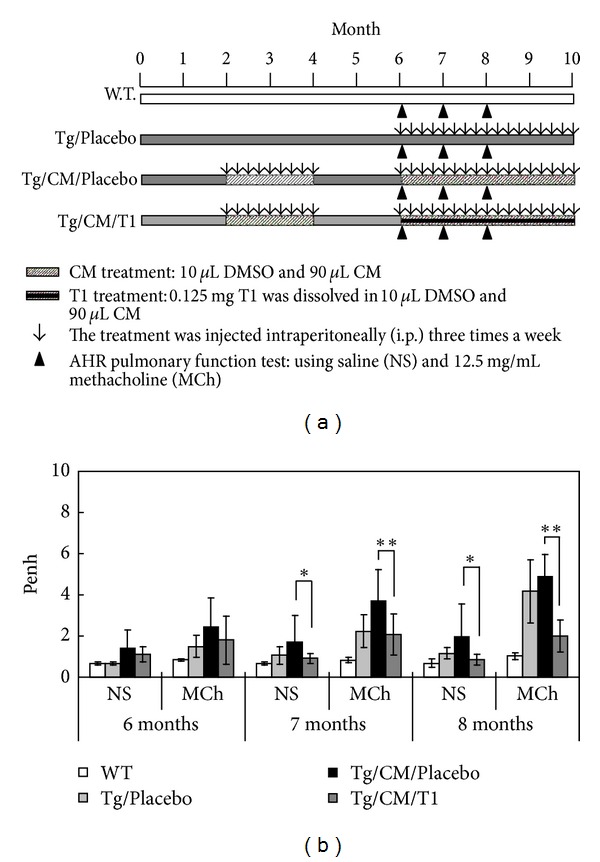
The schedule of animal trials and the effect of tanshinone I on airway hyperresponsiveness (AHR) parameters of pulmonary function. (a) The experimental designation for evidence-based lung tumorigenesis attenuation in animal trial. T1 was injected intraperitoneally (*i.p.*) at 1 mg/kg b.w. three times a week. Mice were sacrificed at 10 months of age after T1 administration for 4 months. (b) The Penh (enhanced pause) is a dimensionless value that represents a function of the proportion of maximal expiratory to maximal inspiratory box pressure signals and of the timing of expiration. Data were presented as mean ± SEM (*n* = 6). The AHR was measured at 6, 7, and 8 months. WT: wild-type FVB mice; Tg/Placebo: hVEGF-A_165_^+/+^ transgenic mice without T1 supplement; Tg/CM/Placebo: condition medium-treated transgenic mice without T1 supplement; Tg/CM/T1: condition medium treated transgenic mice with T1 supplement; NS: normal saline; MCh: methacholine.

A strain of transgenic mice carrying *h* *vegf*-*A*_165_ gene, which induces pulmonary tumor, was used as an *in vivo* lung cancer model for test of T1 therapeutic effects. The animal trials timeline was shown in Figure 3(a). It is well known that AHR is an important measure of pulmonary function, and hyperresponsiveness to methacholine (MCh) is an indicator of lung function. In this study, the AHR was measured at 6, 7, and 8 months. At 6 months, penh values for the wild type, Tg/Placebo, Tg/CM/Placebo and Tg/CM/T1 groups treated with methacholine were 0.84, 1.50, 2.46, and 1.80, respectively; at 7 months, they were 0.82, 2.24, 3.70, and 2.07, respectively; and at 8 months, they were 1.03, 4.17, 4.87, and 2.01, respectively (Figure 3(b)). Thus, the Tg/CM/T1 experimental group treated with 1 mg/kg b.w. of T1 showed significantly lower penh values when compared to Tg/CM/Placebo group (*P* < 0.01). 

### 3.4. Effect of T1 on Pathological Histology

**Figure 4:**
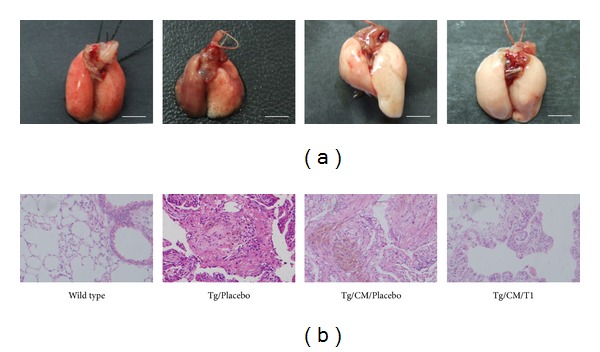
Photographs of whole lungs (a) and stained histopathologic slides of lung tissues (b) from wild-type-FVB mice, hVEGF-A_165_^+/+^ transgenic mice (Tg/Placebo), condition medium-treated transgenic mice (Tg/CM/Placebo), and condition medium treated transgenic mice with T1 supplement (Tg/CM/T1) groups. Tissues from the Tg/CM/T1 group were taken from 10-month-old hVEGF-A_165_ overexpressing transgenic mice after T1 administration for 4 months. The tissue sections were stained with H&E and photographed at 200x magnifications. Scale bar = 5 mm.

Figure 4 and Table 1 showed that various degrees of progressing pulmonary tumors were formed in the transgenic mice and primarily consisted of neoplasms growing on the periphery of the pulmonary alveolus and adenomas growing adjacent to the lung bronchus. In the pulmonary alveolus of the lung bronchus of transgenic mice, some large-grained pink cells were clearly visible. These pink cells were identified as macrophages, which are indicative of an inflammation response (Figure 4). These results show that hVEGF-A_165_ is capable of promoting vascular permeability and an inflammation response. Furthermore, CM treatment alone invoked inflammation and neovascularization as well as promoted lung adenocarcinoma growth (83.3%; 5/6) compared with Tg alone (50%; 3/6), which may be due to various proinflammatory cytokines. Treatment with T1 might have an effect on the balance of proinflammatory and anti-inflammatory cytokines by eliminating the proinflammatory cytokines and then significantly reduced lung tumor growth (16.7%; 1/6) as shown in Table 1.

**Figure 5:**
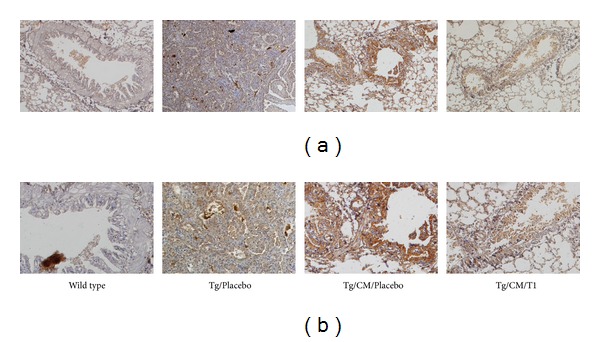
Immunohistochemical (IHC) staining of VEGF expression in lung tissues of the wild-type FVB mice, hVEGF-A_165_^+/+^ transgenic mice (Tg/Placebo), Tg/CM/Placebo, and Tg/CM/T1 groups. Tissues were taken from 10-month-old experimental mice after T1 or PBS administration for 4 months. Sections were stained with primary rabbit monoclonal antibody against human VEGF-A using a 1 : 40 working dilution. Upper panels were observed under 100x magnifications, and lower panels were observed under 200x magnifications.

Angiogenesis is an important factor in the formation of tumors as well as in tumor growth, invasion, and metastasis. VEGF promotes the initial formation of new blood vessels (vasculogenesis) and plays a vital role in the growth and expansion of these new blood vessels (angiogenesis). Using IHC staining, we found that hVEGF was overexpressed in Clara cells of lung tissue from the Tg/Placebo group (Figure 5). Clearly, Tg/CM/Placebo group significantly elevated VEGF overexpression in Clara cells compared with the Tg/Placebo group. Interestingly, the treatment with T1 (1 mg/kg b.w.) significantly blocked VEGF overexpression in Clara cells when compared to the Tg/CM/Placebo group. These results indicate that T1 can reduce hVEGF overexpression, which could in turn eliminate the formation and growth of new blood vessels. This is a possible explanation for how T1 can effectively block tumor growth, invasion, and metastases.

### 3.5. T1 Causes Suppression of Tumor-Formation Signal Genes

**Figure 6:**
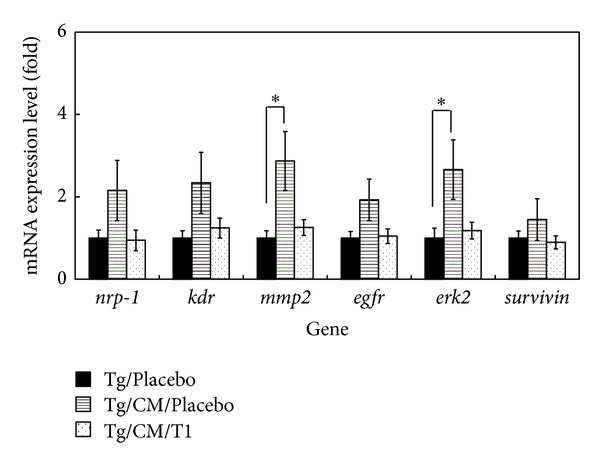
Validations of mRNA expression levels of *kdr*, *nrp-1*, *mmp2*, *egfr*, *erk2,* and *survivin* in the lung tissues of Tg/Placebo, Tg/CM/Placebo, and Tg/CM/T1 mice groups by real-time RT-PCR. A house keeping gene, **β*-actin*, was used as an internal control. The quantitative mRNA expression levels based on three independent repeat experiments of real-time RT-PCR are calculated as mean ± SEM (*n* = 6). *P < 0.05 versus Tg group.

The mRNA expression patterns of* nrp-1*, *kdr*, *mmp2*, *egfr*, *erk2,* and* survivin* in the Tg/Placebo, Tg/CM/Placebo, and Tg/CM/T1 groups were assessed using real-time RT-PCR (Figure 6). T1 did not regulate mCCSP promoter in our internal test (see Figure S1 in Supplementary Material available online at http://dx.doi.org/10.1155/2013/319247); thus T1 directly effected VEGF expression. Furthermore, when compared to Tg/Placebo group, CM treatment alone increased the expression of *mmp2 *(*P* < 0.05), which is believed to be involved in tumor angiogenesis due to its matrix-degrading capacity. However, in this study, treatment with T1 normalized the expression of *mmp2*. In addition, CM treatment alone markedly increased the mRNA levels of *erk2* (*P* < 0.05), which is involved in the regulation of different cellular processes, from apoptosis to cell proliferation and differentiation. We observed a statistically significant decrease in the levels of activated *erk2* in the Tg/CM/T1 group in comparison with Tg/CM/Placebo group.

### 3.6. T1 Causes Suppression of Cell Cycle Progression Signaling Pathways

**Figure 7:**
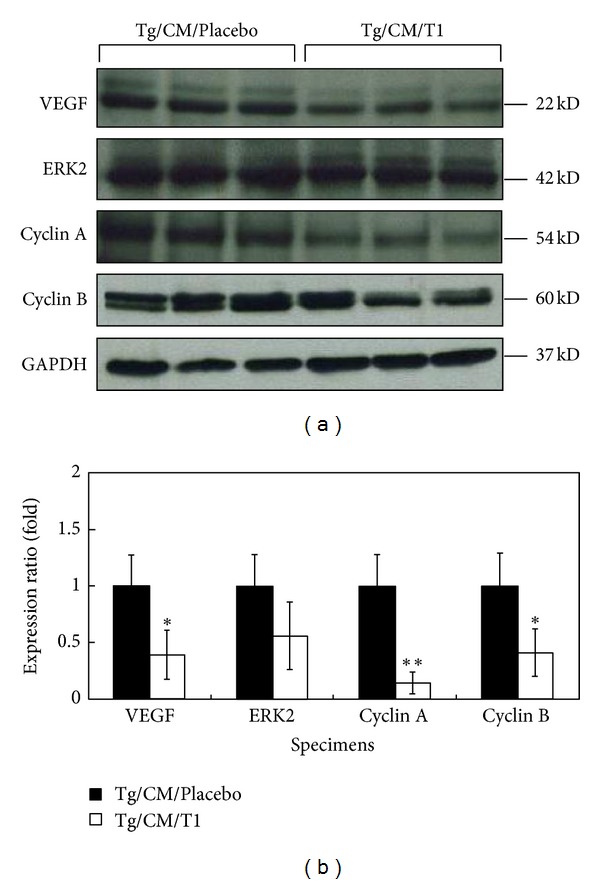
Protein expression levels of VEGF-A, ERK2, Cyclin A, and Cyclin B in the lung tissues of Tg/CM/Placebo and Tg/CM/T1 groups analyzed by western blots (a) and quantitative assay (b). GAPDH was used as an internal control. Protein expression was quantified as mean ± SEM (*n* = 6) from three independent repeat experiments. *P < 0.05 or **P < 0.01 versus Tg/CM/Placebo group.

Western blotting showed that VEGF, Cyclin A, and Cyclin B were downregulated in the Tg/CM/T1 group when compared with the Tg/CM/Placebo group (Figures 7(a) and 7(b)). A possible reason for this cell cycle effect is the reduction of Cyclin A, and Cyclin B that are regulators of the S and G2/M phases, respectively. T1-induced inhibition of Cyclins A and B likely decreases the progression of cells through S and G2/M phases. These results indicate that the proteins involved in the S to M phase transition in Tg/CM/Placebo group were suppressed by T1 treatment. Moreover, we have found that treatment with T1 dramatically decreases tumor formation in 10-month-old hVEGF-A_165_ overexpressing transgenic mice compared with CM treatment alone (Table 1). These results suggest that T1 treatment significantly reduced the tumor formation due to down-regulation of cancer cell cycle.

## 4. Discussion

Earlier studies have shown that Danshen possesses potent anti-inflammatory, antitumorigenic, and antimetastatic effects [17]. T1, one of the major diterpenes isolated from Danshen, shows cytotoxic effects on colon, ovary, lung, and oral cancer cell lines [33]. Furthermore, T1 has been shown to significantly inhibit pulmonary cancer cell migration, invasion, and gelatinase activity *in vitro* and to reduce metastasis, angiogenesis, and tumorigenesis *in vivo* [17]. In this study, we demonstrated that T1 slowed the inhibition of CL1-0 lung cancer cell proliferation by decreasing the expressions of VEGF, Cyclin A, and Cyclin B protein in a dose-dependent manner (Figure 2); thus T1 warrants further development as an antiangiogenic agent in the treatment of lung cancer. Furthermore, Nizamutdinova et al. [34] demonstrated 10 mg/kg T1 did not have any toxic and could markedly inhibited breast tumor growth. Lee et al. [17] revealed 0.3 mg/kg/day T1 significantly inhibited tumor vascularity and fewer tumor metastases in the lung. In addition, we used 10-month-old transgenic mice carrying the mccsp-hVEGF-A_165_-sv40 poly(A) transgene, which overexpresses lung-specific hVEGF-A_165_, to serve as an animal model treatment with 1 mg/kg b.w. T1 to research the regulatory mechanism of T1 in pulmonary cancer. We found that CM treatment alone promoted tumor growth. However, 10-month-old hVEGF-A_165_ overexpressing transgenic mice showed a dramatic decrease in solid tumor formation following a 4-month treatment with T1 three times a week at 1 mg/kg b.w. compared with CM treatment alone. Histological examination also showed that treatment with T1 reduced pulmonary tumor formation and inflammation in hVEGF-A_165_-overexpressing transgenic mice compared with the Tg/CM/Placebo group (Figure 4). Eberly et al. [35] demonstrated that pulmonary function is a predictor of lung cancer mortality. Thus, our results of AHR parameter detections further demonstrated that treatment with T1 in VEGF-A165 overexpressing transgenic mice could effectively reduce pulmonary function damage that may decrease the incidence of lung cancer.

In the initial stages of cancer, cells keep proliferating and leading to both nutrient and oxygen deficiencies that result in a large proportion of cell death. When cancer cells secrete a large amount of VEGF-A_165_, vasculogenesis is induced so as to avail sufficient nutrients and oxygen to the tumor, thus increasing the tumor growth rate [7]. The growth of a solid tumor is dependent on angiogenesis; thus suppression of tumor blood vessel formation offers a new option for the prevention and treatment of cancer. Using IHC staining, we found that T1 reduced VEGF overexpession in Clara cells when compared to Tg/CM/Placebo group (Figure 5). This reduction in VEGF overexpression has an impact on vasculogenesis and angiogenesis and is a vital factor in tumor formation, growth, invasion, and metastasis. Nizamutdinova and his colleagues [36] have shown that T1 effectively inhibited TNF-*α*-induced VEGF production and VEGF-mediated tumor formation. In this study, we also found that treatment with T1 effectively decreased the expression of VEGF, thus demonstrating its utility as an antiangiogenic agent for the treatment of pulmonary cancer.

**Figure 8:**
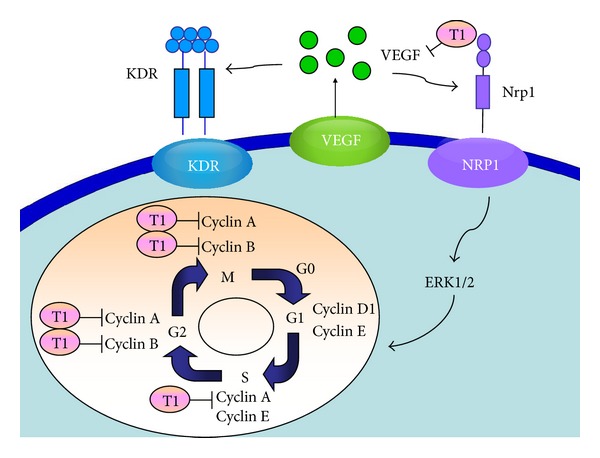
Proposed mechanism of the T1 regulatory pathways in lung tumorigenesis of VEGF-A_165_ overexpressed transgenic mice. The effects of T1 on lung tumor formation are hypothesized to occur through the cell cycle signaling. The diagram shows that T1 might inhibit the expressions of VEGF-A_165_ during signal transduction, as well as reductions of Cyclin A and Cyclin B during S and G2/M cell cycle stages.

Recently, there has been an extensive evaluation of the use of plants and their phytochemicals in the treatment of cancers. In particular, active phytochemicals that function as cell cycle modulators are gaining widespread attention [37]. In this study, we have confirmed that T1 can eliminate pulmonary function damage (Figure 3(b)) and the formation of pulmonary adenocarcinoma (Figure 4 and Table 1). In addition, western blot showed that the expressions of Cyclin A, and Cyclin B in Tg mice were downregulated following CM/T1 treatment as compared with CM/Placebo treatment alone (Figure 7). T1-induced inhibition of Cyclin A and Cyclin B is likely to slow cell cycle progression through the S and G2/M phases. These results indicate that the proteins involved in the transition from the S to M phase in transgenic mice are suppressed by T1. The cell cycle blockage may be the critical mechanism for the observed effects following treatment with T1 (Figure 8). This result suggests that one of the mechanisms of T1 inhibition of pulmonary cancer growth is through inhibition of the tumor-activated cell cycle pathway. A previous study [8] demonstrated that T1 treatment significantly reduced the final prostate tumor weight which was also associated with downregulated cancer cell apoptosis, proliferation, Aurora A protein expression, and angiogenesis.

In summary, T1 significantly reduces lung adenocarcinoma tumor growth. This result may be due to an effect of T1 on the cell cycle and its pathways. This is the first study to demonstrate that T1 inhibits pulmonary tumor formation in animal model through down-regulation of cell cycle at S and G2/M phases. Our research demonstrated that T1 is an efficacious drug candidate for developing a novel class of antipulmonary cancer drugs.

## Supplementary Material

Supplementary figure 1: The RNA expression levels of mouse ccsp gene in the lung tissues of Tg, Tg/CM/Placebo and Tg/CM/T1 groups. (a) Semi-quantitative RT-PCR analysis of mouse ccsp gene mRNA expression in the lung tissues of scarified 10-month-old mice among Tg, Tg/CM/Placebo and Tg/CM/T1 groups. A house keeping gene, **β**-actin, was used as an internal control. (b) The quantification of mouse ccsp gene expression in different experimental mice groups.

## Figures and Tables

**Table 1:** Lung tumorigenesis frequency of wild type, Tg/Placebo, Tg/CM/Placebo, and Tg/CM/T1 groups in the mouse lung tissues (*n* = 6) using histopathological image analysis.

Variable	Wild type	Tg/Placebo	Tg/CM/Placebo	Tg/CM/T1
Normal	100 (100%)	0 (0%)	0 (0%)	2 (33.3%)
Cyst	0 (0%)	2 (33.3%)	0 (0%)	3 (50%)
Damaged alveoli	0 (0%)	3 (50%)	4 (66.7%)	3 (50%)
Mild emphysematous change	0 (0%)	2 (33.3%)	0 (0%)	3 (50%)
Prominent emphysematous change	0 (0%)	3 (50%)	3 (50%)	1 (16.7%)
Hemosiderin-laden macrophages in alveoli	0 (0%)	3 (50%)	5 (83.3%)	1 (16.7%)
Old hemorrhage	0 (0%)	1 (16.7%)	2 (33.3%)	1 (16.7%)
Moderate lymphocytic infiltration	0 (0%)	2 (33.3%)	4 (66.7%)	1 (16.7%)
Marked chronic lymphoid infiltration	0 (0%)	2 (33.3%)	4 (66.7%)	1 (16.7%)
Neoplasm	0 (0%)	1 (16.7%)	1 (16.7%)	0 (0%)
Lymphoma	0 (0%)	1 (16.7%)	1 (16.7%)	0 (0%)
Adenocarcinoma	0 (0%)	3 (50%)	5 (83.3%)	1 (16.7%)
