# Supplementary figures and images for: Active Component of Danshen (Salvia miltiorrhiza Bunge), Tanshinone I, Attenuates Lung Tumorigenesis via Inhibitions of VEGF, Cyclin A, and Cyclin B Expressions
Source: Evid Based Complement Alternat Med. 2013 Apr 9;2013:319247. doi: 10.1155/2013/319247 (PMC3638627; doi:10.1155/2013/319247)

## Supplementary Figure S1

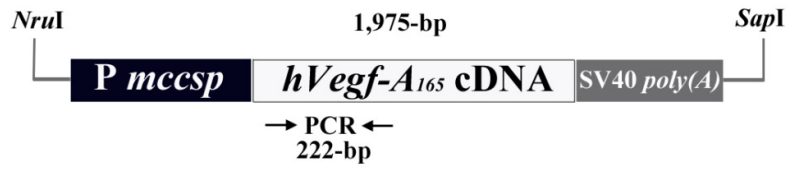

(a)

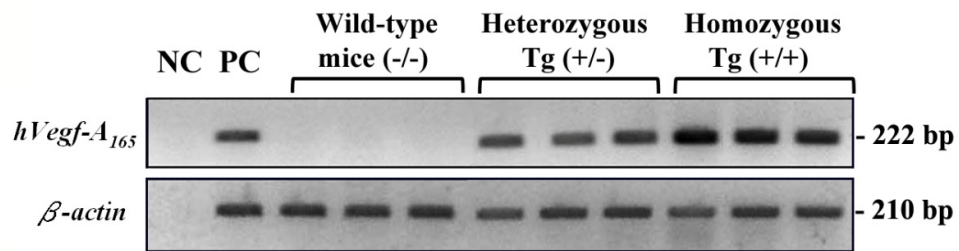

(b)

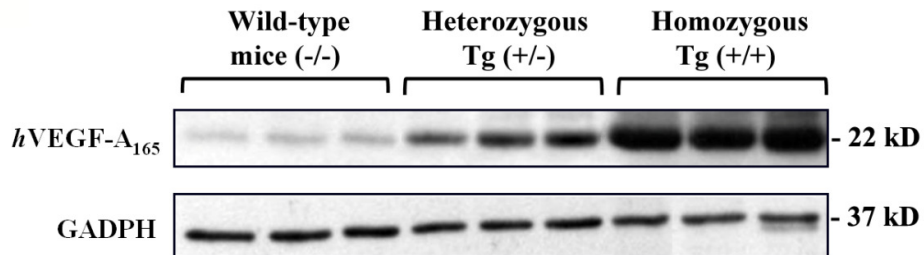

(c)

Supplement: Supplementary file 1 — Supplementary figure 1: The RNA expression levels of mouse ccsp gene in the lung tissues of Tg, Tg/CM/Placebo and Tg/CM/T1 groups. (a) Semi-quantitative RT-PCR analysis of mouse ccsp gene mRNA expression in the lung tissues of scarified 10-month-old mice among Tg, Tg/CM/Placebo and Tg/CM/T1 groups. A house keeping gene, β-actin, was used as an internal control. (b) The quantification of mouse ccsp gene expression in different experimental mice groups. [file 319247.f1.pdf]
